# Interactions of platelets with circulating tumor cells contribute to cancer metastasis

**DOI:** 10.1038/s41598-021-94735-y

**Published:** 2021-07-29

**Authors:** Sina Anvari, Ernest Osei, Nima Maftoon

**Affiliations:** 1 Department of Systems Design Engineering, University of Waterloo, 200 University Avenue West, Waterloo, ON N2L 3G1 Canada; 2 Centre for Bioengineering and Biotechnology, University of Waterloo, Waterloo, ON Canada; 3 Department of Medical Physics, Grand River Regional Cancer Centre, Kitchener, ON Canada; 4 Department of Physics and Astronomy, University of Waterloo, Waterloo, ON Canada; 5 Department of Clinical Studies, Ontario Veterinary College, University of Guelph, Guelph, ON Canada

**Keywords:** Computational biophysics, Cancer models, Metastasis

## Abstract

Recent studies have suggested that platelets have a crucial role in enhancing the survival of circulating tumor cells in the bloodstream and aggravating cancer metastasis. The main function of platelets is to bind to the sites of the damaged vessels to stop bleeding. However, in cancer patients, activated platelets adhere to circulating tumor cells and exacerbate metastatic spreading. Several hypotheses have been proposed about the platelet–cancer cell interactions, but the underlying mechanisms of these interactions are not completely understood yet. In this work, we quantitatively investigated the interactions between circulating tumor cells, red blood cells, platelets, plasma flow and microvessel walls via computational modelling at the cellular scale. Our highly detailed computational model allowed us to understand and quantitatively explain the role of platelets in deformation, adhesion and survival of tumor cells in their active arrest to the endothelium.

## Introduction

Platelets are one of the blood components which contribute to the wound healing and hemostasis process by initiating a blood clot. Platelets have no nucleus, they have a round or oval shape, and their diameter is about two micrometers which is 2–3 times smaller than that of red blood cells (RBCs)^1^. Platelets live around eight to ten days and the normal platelet count is between 150,000 to 450,000 platelets per microliter of blood which is approximately one-tenth of the number of RBCs^2^. The main role of platelets is to stop bleeding when the blood vessels get damaged, by gathering together to form blood clots^3^.

Trousseau established the link between the platelets and cancer in the nineteenth century^4^. He observed excessive blood clotting in patients diagnosed with cancer including himself^5^. It is now well recognized that within the first stage of tumor growth, factors are secreted that stimulate and increase the production of platelets that can lead to thrombocythemia^5^. Both high platelet count and expression of coagulation factors by tumor cells are negative prognostic markers in different types of cancer^5,6^. However, during the metastatic stage and chemotherapy, the platelet count decreases^5,7^. Platelets adhere to circulating tumor cells (CTCs) with receptor-ligand bonds and the attached platelets facilitate adhesion of the CTC to the vessel wall^5,8^.

Cancer studies suggested the role of platelets in metastasis^5^. When tumor cells detach from the primary tumor and intravasate into the blood vessels, there is a high probability that they will be destroyed due to high shear stress exerted by the blood flow and the endothelium lining the blood vessels, or due to the immune response of the body^9^. However, it has been suggested that because the platelets adhere to CTCs, immune cells cannot recognize CTCs and as a result, the survival rate of CTCs increases^8,10^. Additionally, platelets can transfer the major histocompatibility complex to CTCs, which cause CTCs to mimic host cells and confound the immune cells^9^. Nieswandt et al.^11^ demonstrated that aggregation of platelets around CTCs inhibits the protective activity of immune cells. Therefore, minimizing the platelets–CTC microthrombi formation will lead to the attenuation of metastasis^10,12^. Additionally, even if leukocytes recognize CTCs by any chance, they do not have access to destroy them because platelets act as a barrier in front of them^8^. Moreover, the platelet shield around CTCs has been proposed to protect CTCs from shear stress by reducing the exerted force^13^. Furthermore platelets are able to induce several factors (such as platelet-derived growth factors) which can stimulate and accelerate epithelial to mesenchymal transition in CTCs^14,15^. Thus, CTC–platelets interactions can lead to more efficient migration and easier extravasation out of the circulatory system^11,12,16^.

It was demonstrated that there is a higher probability that a CTC that is surrounded by platelets adheres to the vessel wall^17^. The activation of the platelet endothelial cell adhesion molecule-1 (PECAM-1, CD31), that modulates the junctions between adjacent endothelial cells and regulates the transendothelial migration of CTCs, is an additional help for firm adhesion and extravasation of CTCs^18,19^. Stoletov et al. proposed that, during intravasation, CTCs secrete vascular endothelial growth factor (VEGF) which results in the permeabilization of microvessels which expand the space between two neighboring endothelial cells^20^. Knowing that platelets can also secrete VEGF, CTC–platelet interaction increases the possibility of successful extravasation due to the higher permeability of microvessels^8^.

There is solid experimental evidence that thrombocytopenia caused by either platelet depletion with anti-platelet drugs or by defective platelet production significantly reduces the metastatic tumors. Borsig et al.^16,21^ showed that heparin treatment attenuates metastasis in mice by inhibiting selectin- and integrin-mediated interactions of platelets with carcinoma cell-surface ligands. By comparing two groups of mice, they proved that a single injection of heparin can impair CTC–platelet interactions and greatly reduce metastasis^21^. Inhibiting CTC–platelet interaction leading to reduced metastases was further studied experimentally using aspirin^22^, prostacyclin (${PGI}_{2}$)^23^ and dipyridamole^24^.

In addition to experimental studies, physics-based computational models can help us understand different steps of the metastasis cascade and interpret experimental and clinical observations as were comprehensively reviewed before^25^. The interactions between CTCs, platelets, and endothelial cells mainly rely on the cell–cell adhesion bonds. Almost all computational studies about CTC adhesion to the vessel wall have used spring-based models for simulating the adhesion forces that was originated from. An initial simple mathematical model by Bell and coworkers that described the adhesion process by employing a thermodynamic approach^26^. Later, a more comprehensive mathematical model was proposed which simulated all phases of the adhesion process^27^.

Although contributions of platelets in cancer metastasis have been studied in recent years, the underlying mechanisms that initiate and support the interactions between CTCs and platelets are still not well understood. Observing interactions between platelets, CTCs, and endothelium in vivo has not been possible experimentally so far and although there were suggestions of roles of platelets in metastasis, there has not been any quantitative study that could show the phenomena involved in the process. In this work, we used a computational model based on first principles and a priori knowledge to study the motions of platelets and CTCs in the plasma flow and their interactions with one another, RBCs, and the vessel wall to better understand the underlying mechanisms of metastasis. Our computational model considers blood plasma as an incompressible fluid solved using the three-dimensional Lattice-Boltzmann Method (LBM) and considers cells as deformable bodies modelled using a coarse-grained spectrin link membrane approach. The Immersed Boundary Method (IBM) was used to couple the fluid solver with the coarse-grained model and it enabled modelling of different biophysical interactions at mesoscale including the transport of CTCs in the microvasculature and receptor-ligand interactions between CTC, platelets, and endothelial cells. The arrest of circulating tumor cells can potentially be the results of geometrical factors (e.g., the diameter of microvessel and diameter of CTCs) and blood flow patterns suggested by Ewing^28^. As suggested by Paget^29^ in “seed and soil” theory, the interactions of CTCs with the microenvironment can be another cause of CTC arrest and attachment to the microvessel wall. In this study we focus on the arrest of CTCs due to active interactions between a CTC, platelets and endothelium and not geometrical factors. Our observations, consistent with previous experiments on the platelet-aggregation inhibitor drugs, highlight the role of platelets in cancer metastasis and especially the arrest and extravasation process of CTCs.

## Results

### Rolling of circulating tumor cells causes formation of localized vortex

**Figure 1:**
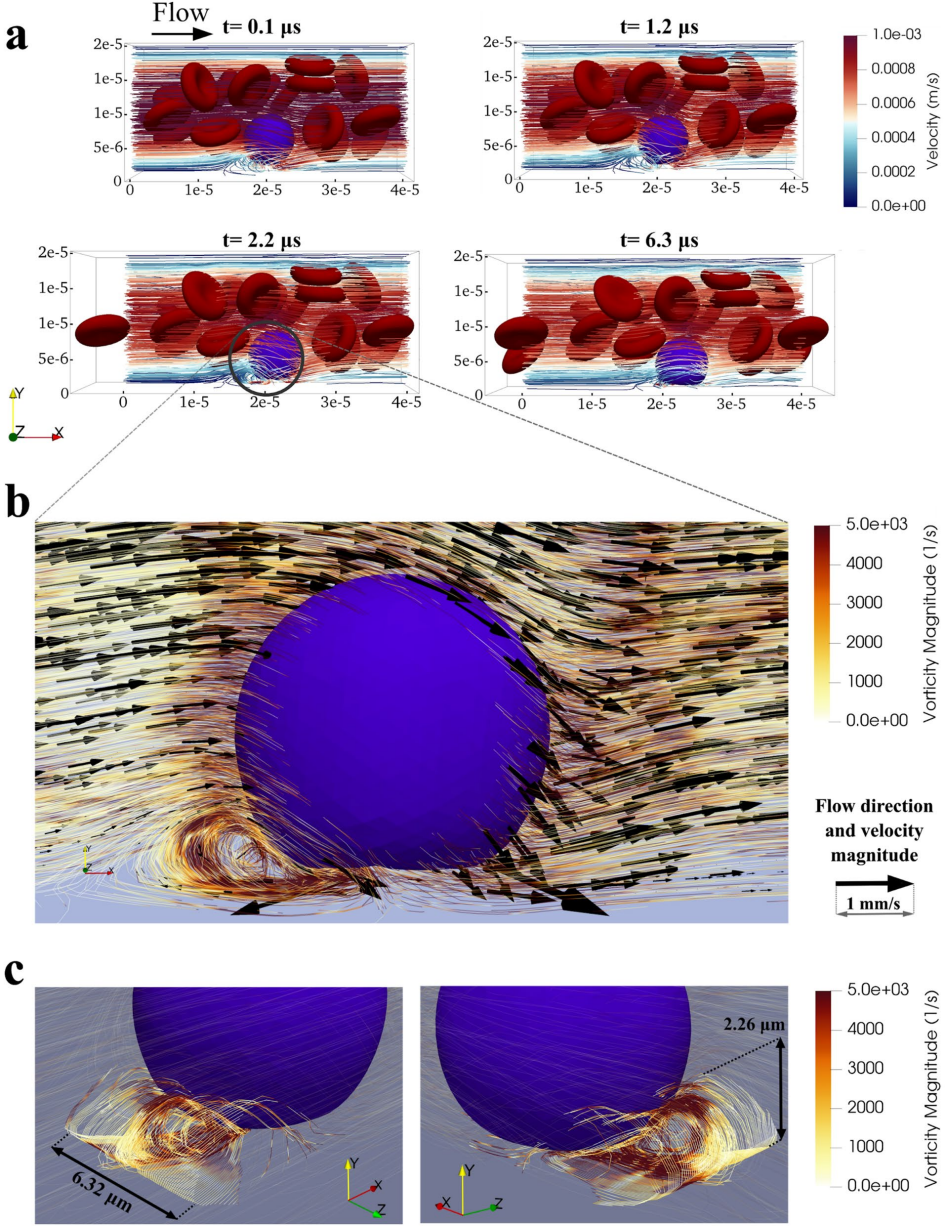
Rolling motion of the CTC in the vicinity of the microvessel wall with a focus on the localized vortex that is formed upon the rolling motion. (**a**) Rolling dynamics of the CTC with no attached platelets at four initial instances of rolling. When CTC gets closer to the vessel wall, a localized vortex forms in the plasma flow behind the CTC in the vicinity of the vessel wall; (**b**) Closer view of the CTC and vortex at t = 2.20 μs with more quantitative details on the magnitude and direction of the velocity and the magnitude of the vorticity; (**c**) Visualization of the vortex tube in 2 3-D views. The maximum length and maximum height of the vortex tube are indicated in the figure. Although the seed points of the streamlines are distributed uniformly along a line source, the concentration of the streamlines in the middle part of the vortex tube, consistent with high vorticity magnitude, causes high shear rates in the region which may lead to activation of the adhesion molecules on attached platelets and initiation of CTC–platelets interactions.

Our computational model showed the formation of a localized vortex behind the CTC at the onset of the rolling motion. Figure 1a shows the plasma flow and motion and deformation of a CTC at four instances of the rolling motion (Supplementary Video S1 also shows the rolling motion of CTC with no attached platelets). A closer view at the flow streamlines in the vicinity of CTC and vessel wall (Fig. 1b, Supplementary Video S2) shows a localized vortex behind the contact area of CTC and endothelial cells. Considering the Poiseuille flow profile expected for the plasma flow inside microvessels, the velocity of the fluid should be around 0 near the vessel wall (Supplementary Fig. S1c of the Supporting Information) but, due to the presence of the vortex, the fluid velocity reaches to 2.57 mm/s that is twice the highest Poiseuille velocity magnitude in the microvessel (on the centreline). Thus, the localized vortex leads to a high shear rate around the CTC near the vessel wall and therefore may initiate the activation of adhesion molecules that attract platelets around the CTC. Karino et al. demonstrated with an in vitro experiment that the localized vortex can activate the platelets and intensify the adhesion properties of the platelets^30,31^. Localized vortex can also force an outward migration of platelets that exist inside the vortex^32^ and push them toward the CTC. In addition, the vortex causes a sudden flow reversal that is likely the basis for the increased permeability of the endothelium and increased adhesion and extravasation probability^33^.

The vortex formed upon the onset of the rolling motion and the maximum vorticity magnitude of 4720 s^−1^ occurred at 2.20 μs after the onset of the rolling motion of CTC (Fig. 1a). Gou et al. used a computational model to show how localized vortex at the branching point of the microvessel can regulate cell–cell interactions and can result in increased CTC adhesion^34^. Here we showed the vortex formation in the CTC rolling motion in a microvessel for the first time. The localized vortex can result in higher shear forces in that region and the stimulation of CTC–platelets interactions.

Figure 1c shows the vortex tube from different views. The maximum dimensions of the vortex tube reached to 6.32 μm in length and 2.26 μm in height. For visualizing the vortex tube in Fig. 1c, the seed points of the streamlines were distributed uniformly on a line source in the vicinity of the localized vortex. However, the streamlines were concentrated in the middle part of the vortex tube (Fig. 1c) which indicates the higher vorticity magnitude and higher shear rates in the region which can stimulate adhesion molecules on the platelets and activate the surrounding platelets^30^.

### Platelets reduce time and distance of circulation of circulating tumor cells by enhancing adhesion

To examine the effect of platelets on the adhesion and arrest of CTC, we simulated the movements of CTC in a microvessel as detailed in “Materials and methods”. Considering the low abundance of CTCs in the blood, all the simulations were done using only one CTC to speed up the calculations. We studied the effect of platelets on the dynamics of CTC near the vessel wall by changing the number of platelets in the model from 0 to 15 (at a 5-platelet increment). The model included RBCs but other far less abundant blood cells, such as leukocytes, monocytes were not modeled. All simulations were carried out with the same initial and boundary conditions except for the number of platelets around the CTC.

Because of the physical similarities between the CTCs and white blood cells (i.e. shape, size, and stiffness), the margination of CTC is quite similar to the margination of white blood cells^35,36^. Therefore, we skipped the margination movement to focus more on adhesion and arrest of the CTC by setting the initial position of the CTC near the vessel wall in all simulations to reduce the time required for the CTC to marginate to the vessel wall.

**Figure 2:**
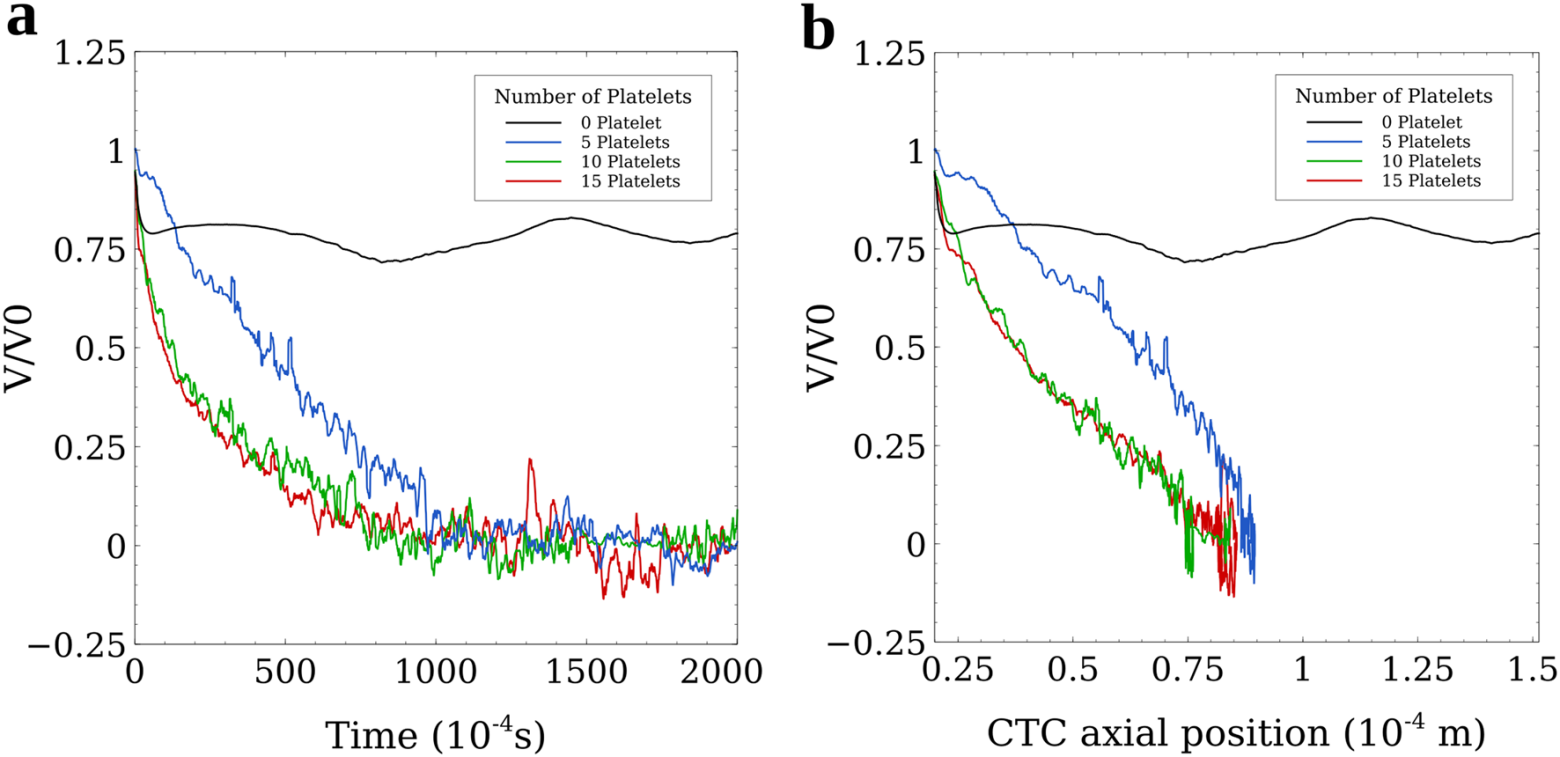
*Velocity–Time* and *Velocity–Axial Position* graphs showing that CTC–platelets interactions significantly enhance the formation of the firm adhesion bonds between CTC and the endothelial cell. (**a**) *Velocity–Time* graph showing the effect of the number of attached platelets on the adhesion of CTCs. With increasing the number of platelets attached to the CTC, the velocity of the CTC decreases faster and the firm adhesion of the CTC to the vessel wall, as a prerequisite for extravasation, occurs in a shorter time. Lodging of the CTC to the vessel wall saves the CTC from further circulation in the vascular system, exposure to the high shear stress in larger vessels, and encountering immune cells. As a result, the faster the CTC adheres to the vessel wall, the higher survival chance it has; (**b**) *Velocity–Axial Position* of CTC graph showing the distance the CTC rolls and crawls motions in the microvessel until the firm adhesion occurs. Increasing the number of attached platelets reduces the rolling distance which leads to less shear forces exerted on the CTC by endothelial cells and as a result, chances of CTC survival and extravasation increases.

The results of the simulations show that upon margination, the CTC will immediately start a rolling motion due to the CTC-wall adhesion forces. As Fig. 2 shows, when there was no platelet around the CTC (black line), the CTC continued its rolling motion without any remarkable changes in its velocity. This rolling motion continued as long as we continued the simulation. Increasing the number of platelets attached to the CTC, slowed down the rolling motion of the CTC eventuating to a firm adhesion between the CTC and vessel wall. The *Velocity–Time* graph of Fig. 2a shows that increasing the number of platelets attached to the CTC, decreased the duration of the rolling motion and caused the firm adhesion to happen faster. For the CTC with 5 platelets attached to it, the firm adhesion took almost 0.1 s to happen but when the number of attached platelets increased to 15, the adhesion time decreased by 0.06 s (a 40% decrease). Furthermore, the *Velocity-Position* graph of Fig. 2b shows the rolling distance until firm adhesion for the CTC with 15 attached platelets was up to 8% shorter in comparison with that observed for a CTC with 5 platelets attached to it (Supplementary Videos S3 and S4 show the adhesion dynamics of the CTC with 5 and 10 attached platelets, respectively).

Another information that is provided by Fig. 2a,b is that the adhesion of CTC to the vessel wall with 10 attached platelets is similar to the adhesion of CTC with 15 attached platelets. Considering that only the platelets existing on the contact area between the CTC and the vessel wall can affect the adhesion dynamics, we can conclude that for an 8 μm-diameter CTC, 10 platelets can cover the entire contact area on the CTC. Although a higher number of attached platelets has no significant effect on the time and distance of the firm adhesion of CTC to the vessel wall, these platelets can shield CTC from external mechanical forces and preserve the integrity of the CTC. It should be noted that at the stage of firm adhesion, the velocity of CTCs still has small oscillations around 0 (Fig. 2a,b) because of the forces applied by the plasma flow and RBCs and the spring-like probabilistic adhesion bonds, but the change in the position of the CTC remains negligible (less than 5 µm).

### Platelets preserve the integrity of circulating tumor cells

We studied the effects of platelet shield on the CTC deformation and quantified these effects using Taylor’s deformation parameter of aspect ratio^37^. Taylor’s aspect ratio is a dimensionless quantity defined as $\varsigma =\frac{L-B}{L+B}$; where L and B are major and minor axes of the ellipsoid (CTC) with the same moment of inertia. $\varsigma$ is originally defined for small distortions from the spherical form that occur at small velocities of the flow such as plasma flow in microvessels. Taylor’s aspect ratio is 0 for a perfect sphere. The highest aspect ratio in our model is 0.133 that belongs to the rolling CTC with no attached platelet and the experimental results show that the Taylor’s deformation parameter of aspect ratio is an accurate measure of deformation when the values of $\varsigma$ are less than 0.4^37^, which is the case in our simulation.

**Figure 3:**
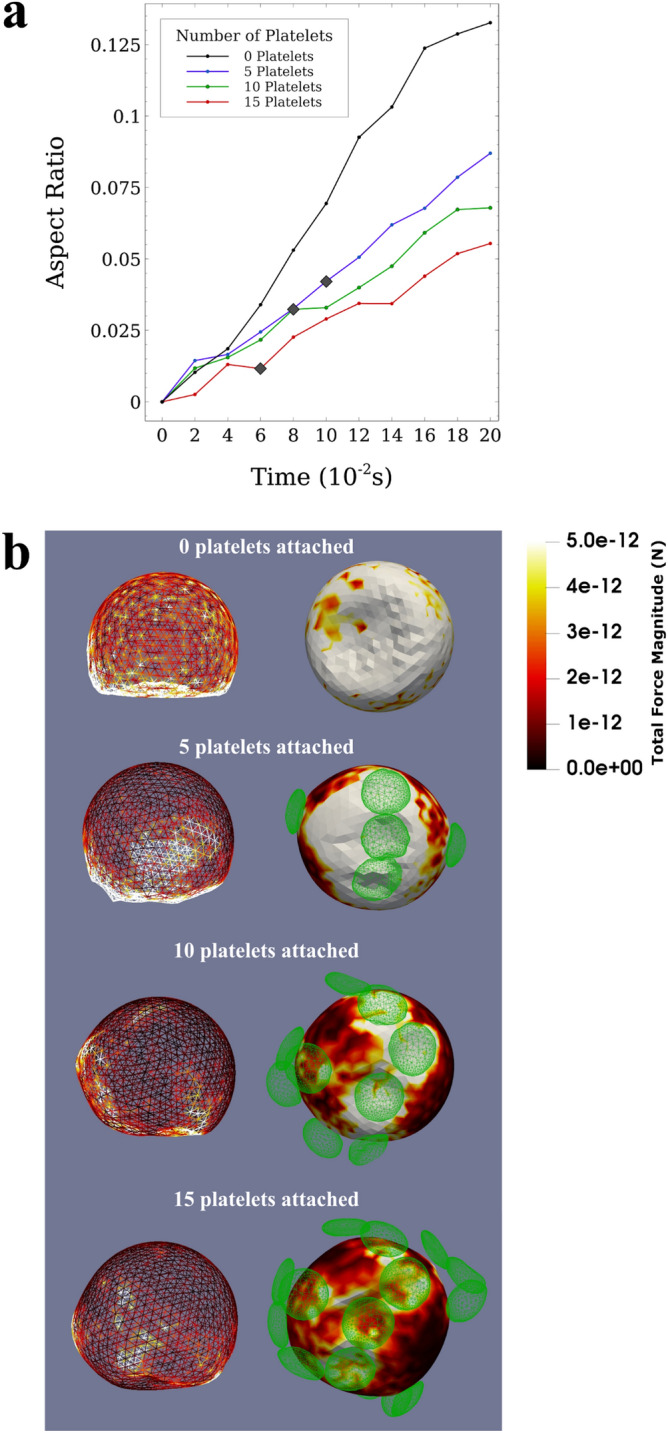
The deformation of the CTC as a function of the number of attached platelets and the force distribution on the membrane of the CTC. (**a**) Evolution of the *aspect ratio* with time shows the deformation of CTCs over the simulation time for different numbers of attached platelets. The aspect ratio is calculated using $\varsigma =\frac{L-B}{L+B}$. Also, the firm adhesion instance is indicated with grey diamond for the cases in which there are attached platelets and the firm adhesion occurs. The graph shows that the attached platelets can reduce the aspect ratio (i.e. deformations) of the CTC with no attached platelet to less than half when 15 platelets are attached to the CTC. Attached platelets can preserve the integrity of the CTC and reduce the deformations of the CTC by making the force distribution on the CTC membrane homogeneous; (**b**) Front view (on the left side) and bottom view (on the right side) of the CTC showing its shape deformation in the rolling motion (for the CTC with no attached platelets, top) and in firm adhesion state (for CTCs with 5, 10, and 15 attached platelets). The membrane is coloured with the distribution of the magnitude of the total force on the CTC. External forces applied on small areas of CTC membrane can damage the CTC during the adhesion process. Platelets attachment to the CTC reduces the maximum force applied to the CTC by making the force distribution on CTC increasingly homogeneous.

Figure 3a shows the evolution of the aspect ratio of a CTC with an increasing number of attached platelets up to 0.20 s after the onset of the rolling motion. The simulation time was specifically chosen to provide adequate time for the CTC to adhere to the vessel wall. We continued the simulations for an additional 0.20 s to guarantee the stability of the results (see Time step). At the onset of the rolling motion, the aspect ratio of all CTCs was zero indicating spheres. During the rolling motion and after the firm adhesion instance (indicated in Fig. 3a with grey diamond), the aspect ratio of all CTCs consistently increased with time. The slope of the increasing trend of the aspect ratio of CTCs, decreased with the number of attached platelets from 0.67 ${\mathrm{s}}^{-1}$ for the CTC with no platelet to 0.28 ${\mathrm{s}}^{-1}$ for the CTC with 15 attached platelets. At 0.20 s after the onset of the rolling motion, the CTC with no attached platelets was in rolling motion and its aspect ratio was 0.13 while a firm adhesion was formed between the vessel wall and the CTC with 5 attached platelets, and its aspect ratio decreased to 0.087. Increasing the number of attached platelets to 10 and 15 decreased the aspect ratio to 0.068 and 0.055 (at 0.2 s after the onset of the rolling motion) which are almost half and less than half of the aspect ratio of the CTC with no attached platelets, respectively. Our results demonstrate that increasing the number of platelets around the CTC (enlarging the platelet shield) reduces the deformation of the CTC in the adhesion process.

The total force distributions on the CTC membrane in the firm adhesion state (Fig. 3b) also confirm the protective role of platelets against external forces applied on the CTC. The force distributions in the firm adhesion state in these figures show that the maximum force applied to any individual node decreases as the number of attached platelets increases (from 328 pN with 5 platelets to 48.9 pN and 28.7 pN for 10 and 15 platelets, respectively). Also, the integral of the force magnitude over the entire CTC in the firm adhesion state decreased from 14.5 nN with 5 platelets attached to 10.6 nN and 7.55 nN for 10 and 15 platelets attached to the CTC respectively. Reduction in the detrimental force can be due to the wider contact area between the CTC and vessel wall as the results of attached platelets that makes the distribution of force on the CTC membrane homogeneous. Mechanical experiments of the rupture of the cell membrane^38,39^ showed that external forces in the order of 10–30 nN are required to rupture the cell membrane. Our results suggest that attached platelets reduce the chance of CTC lysis due to external forces.

It should be noted that for the case where there is no platelet attached to the CTC, the firm adhesion bond did not form between the CTC and the endothelial cells as long as we could continue the simulation, and the CTC continued its rolling motion. Consequently, the forces applied on the CTC membrane (without attached platelets) in the rolling motion (with the maximum of 170 pN and the integral of 13.4 nN over the entire membrane) were not compared to the forces CTCs with attached platelets experienced with the firm adhesion bonds to the endothelial cells.

### Wall shear stress increases during adhesion and arrest of circulating tumor cells

**Figure 4:**
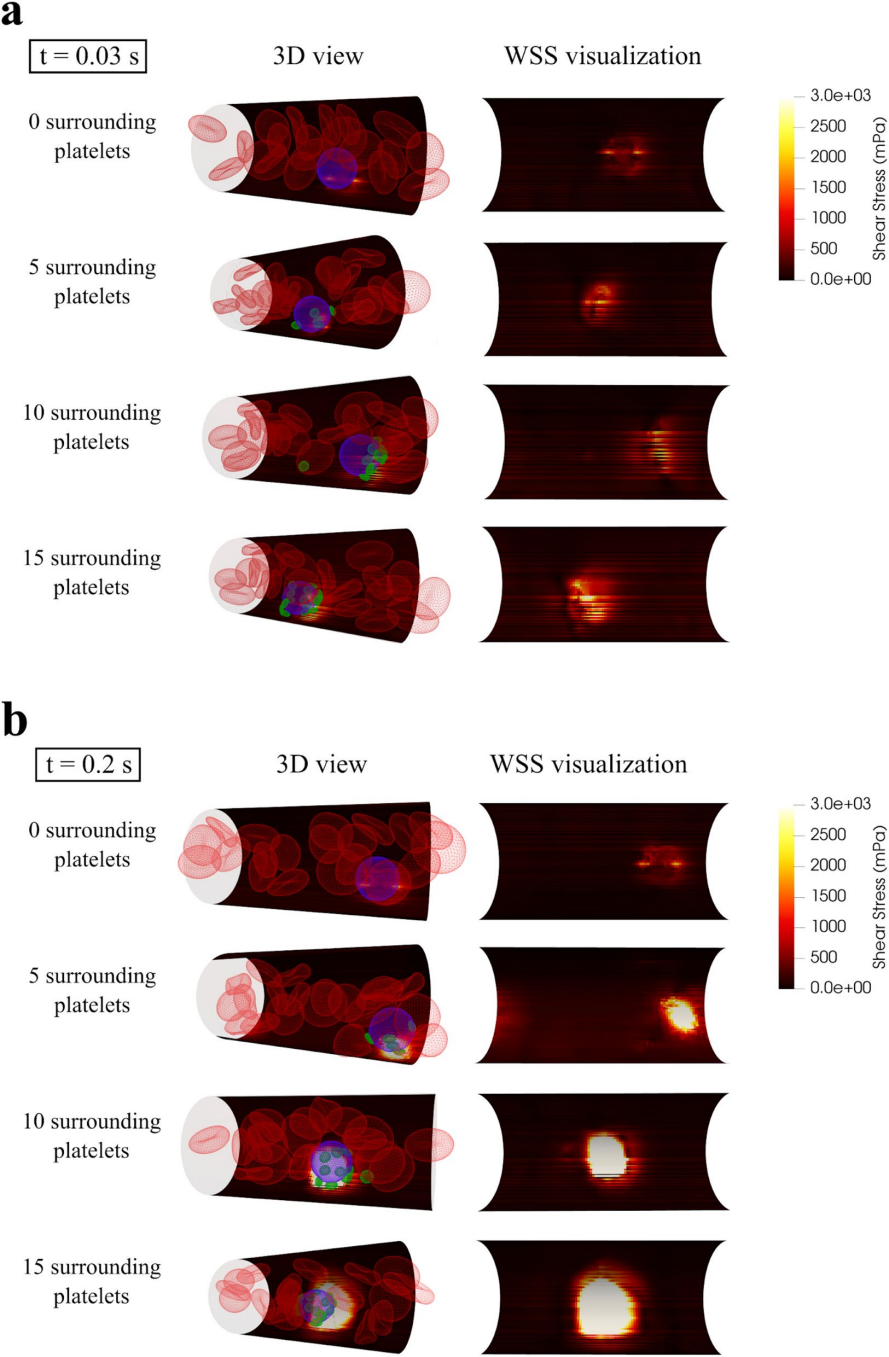
The wall shear stress (WSS) during rolling state and firm adhesion of the CTC. (**a**) Illustration of WSS on the microvessel wall as a result of rolling motion of CTCs and the attached platelets near the vessel wall at t = 0.03. The area of elevated shear stress expands when more platelets are attached to the CTC. High shear stress on the endothelial cells initiates the adhesion molecules that is a necessary step before firm adhesion occurs; (**b**) Illustration of the WSS on the microvessel wall for different numbers of platelets attached to the CTC at t = 0.20. When there is no platelet attached to the CTC, the CTC continues its rolling motion and the WSS is almost the same as the one at t = 0.03 (**a**). When there are platelets attached to the CTC, the firm adhesion occurs and the WSS substantially increases in comparison with rolling motion (**a**) and reaches to its maximum magnitude. Additionally, the area of the elevated WSS expands increasingly by increasing the number of attached platelets.

The expression of VEGF is highly dependent on the wall shear stress (WSS) magnitude^40^ and elevated VEGF can facilitate extravasation by expanding the space between two neighboring endothelial cells^17^. Figure 4a shows that at t = 0.03 s when all CTCs (with any number of attached platelets) are in the rolling state, the endothelium experiences at least 200% higher WSS in a small area in the vicinity of CTC. The area of the elevated WSS expands with increasing the number of attached platelets which resulted in expanded contact area between CTC–platelets complex and the endothelium and is consistent with easier firm adhesion formation in terms of time and distance of travel as described above.

Figure 4b illustrates the WSS at t = 0.20 s, when the firm adhesion has occurred for all CTCs with attached platelets but the CTC with no attached platelet is still in the rolling motion phase. In the firm adhesion state of CTCs with attached platelets, the WSS increases substantially (more than 100%) in comparison with the rolling state of the same CTC. In contrast, since the CTC with 0 attached platelets does not form firm adhesion with the endothelial cells and continues its rolling motion, the WSS at t = 0.20 s has the same value as it had at t = 0.03 s. Additionally, the area of the endothelium experiencing elevated WSS (greater than 1 Pa) also expanded as shown in Fig. 4b from 1.25 μm^2^ around CTC with no platelets to 17.3 μm^2^, 52.8 μm^2^, and 104 μm^2^ with 5, 10 and 15 platelets, respectively.

**Figure 5:**
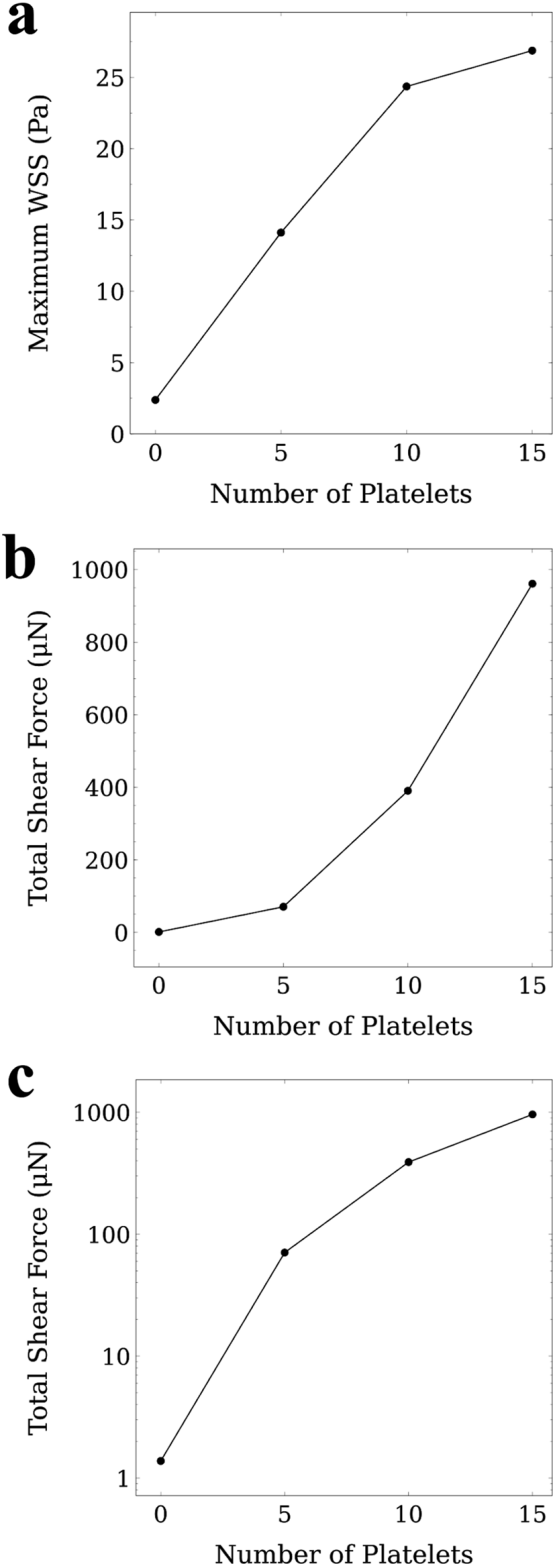
Maximum wall shear stress (WSS) and total shear forces applied to the endothelial cells as functions of numbers of platelets attached to the CTC. (**a**) Maximum WSS applied to the endothelial cells because of the presence of the CTC and the attached platelets in vicinity of the vessel wall. The maximum WSS increases with increasing the number of attached platelets. Because the contact area between the platelets attached to the CTC, and the endothelial cells reaches to its maximum value with a critical number of platelets (here 10 platelets), the maximum WSS remains almost the same after the critical number of attached platelets (10 platelets); (**b**) Total shear force applied to the endothelial cell in the vicinity of the CTC and attached platelets. The total shear force applied to the endothelial cells is calculated by integrating the WSS over the contact area at t = 0.20 s. By increasing the number of attached platelets, the total shear force increases substantially which indicates a higher chance of VEGF expression and CTC extravasation; (**c**) When the total shear force plotted logarithmically, it shows the same trend as the maximum WSS applied to the endothelium (**a**).

Figure 5a shows the maximum WSS occurred during the simulation. The maximum WSS of 2.38 Pa was observed in the vicinity of the CTC in rolling motion with no attached platelets. In presence of 5 attached platelets, the maximum WSS reaches 14.1 Pa which is approximately 6 times its value for the rolling CTC with no platelet attached. Increasing the number of attached platelets around the CTC to 10 and 15, increases the maximum WSS to 24.4 and 26.9 Pa respectively. Consistently, the total shear force exerted on the endothelium by the CTC compound (obtained by integrating the WSS over the contact area at t = 0.20 s) constantly increases by increasing the number of platelets as it is shown in Fig. 5b. Interestingly the total shear force plotted logarithmically in Fig. 5c shows the same increasing trend as the WSS in Fig. 5a. While a CTC in rolling motion with no platelets attached can cause a shear force of 1.38 μN on the adjacent endothelial cells, a CTC with 5 attached platelets in firm adhesion can cause a shear force of 70.7 μN on the adjacent endothelial cells and as Fig. 5b shows the applied shear force increased by increasing the number of attached platelets. The drastic jump in the shear force due to firm adhesion to the endothelial cells can boost the VEGF expression in that region and increase the chance of CTC extravasation.

### Softer and smaller CTCs adhere more effectively to the vessel wall

**Figure 6:**
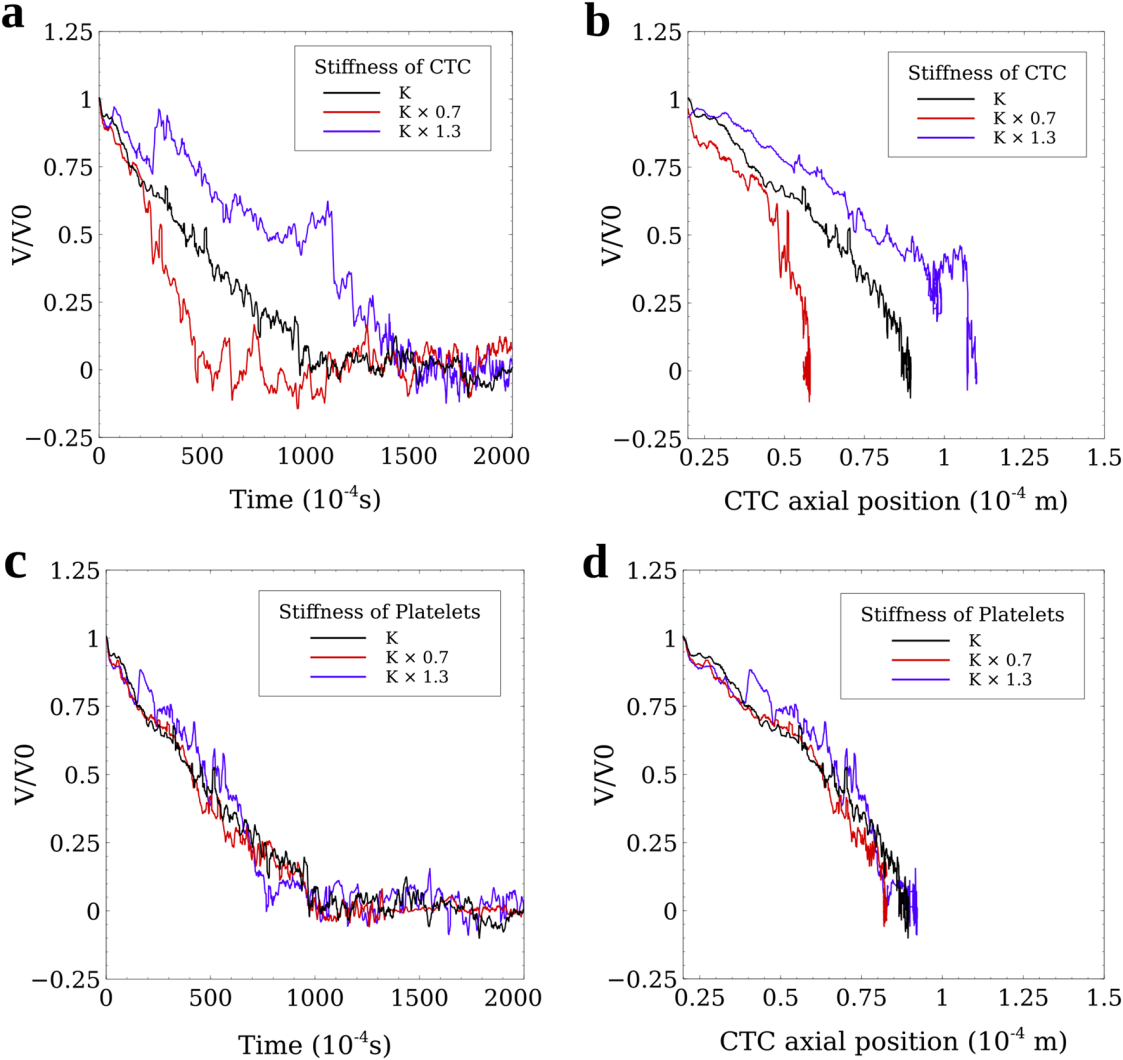
Analysis of the effect of the stiffness of the CTC and platelets on the adhesion and arrest of CTCs. A CTC with 5 attached platelets floating in the blood flow was investigated. (**a**) Softer CTCs adhere faster to the vessel wall than stiffer CTCs. A CTC by 30% softer (red line) leads to 50% decrease in the adhesion time while a 30% stiffer CTC causes a 50% increase in the adhesion time. Faster adhesion of softer CTCs is mainly because of the expanded contact area between CTC and the vessel that leads to a higher number of adhesion bonds between the CTC with attached platelets and the endothelial cells; (**b**) Softer CTCs travel shorter distances before firm adhesion occurs, mainly because they adhere faster than normal tumor cells; (**c**) The adhesion time of the CTC with attached platelets that are 30% softer or stiffer than the baseline platelets, does not have a significant effect on the adhesion time and the CTCs in all the three cases form the firm adhesion at the same time; (**d**) Softer platelets (by 30%) can decrease the rolling distance of the CTC by 8% but 30% stiffer platelets do not cause any remarkable difference on the rolling distance of the CTC compared to the baseline platelets. Altogether, the stiffness of the CTC is a more determining factor in the adhesion and arrest of the CTC than the stiffness of the attached platelets.

To study the effect of CTC stiffness on its adhesive properties, we repeated the simulation with 5 attached platelets. Katsantonis et al. suggested that the malignant transformation of tumor cells reduces F-actin in the cell cytoskeleton by ~ 30% and leads to higher deformability of the invasive cancer cells^41^. Thus, we studied the effects of increasing and decreasing the stiffness of CTCs by 30% through changing the spring constants related to each mechanical behavior of the CTC membrane explained in “Materials and methods”. As Fig. 6a,b illustrate, softer CTCs adhered 0.05 s faster (equivalent to 50% decrease in the adhesion time) to the vessel wall. We showed that invasive CTCs, that are known to be softer than normal tumor cells^41–43^, deform and adhere to the vessel wall more efficiently^44^. Our results also show that stiffer CTCs need 0.05 s more time to adhere to the vessel wall that is 50% longer than the time that a baseline CTC takes. This phenomenon can be due to the smaller contact area between the stiffer CTC and the vessel wall.

Additionally, we performed the simulation with 30% stiffer and 30% softer platelets using the same approach described for CTCs. As Fig. 6c,d show, changing the mechanical properties of platelets does not have remarkable effects on the adhesion and arrest of the CTC. Thus, the results of the simulation are not sensitive to the mechanical properties of the platelets.

**Figure 7:**
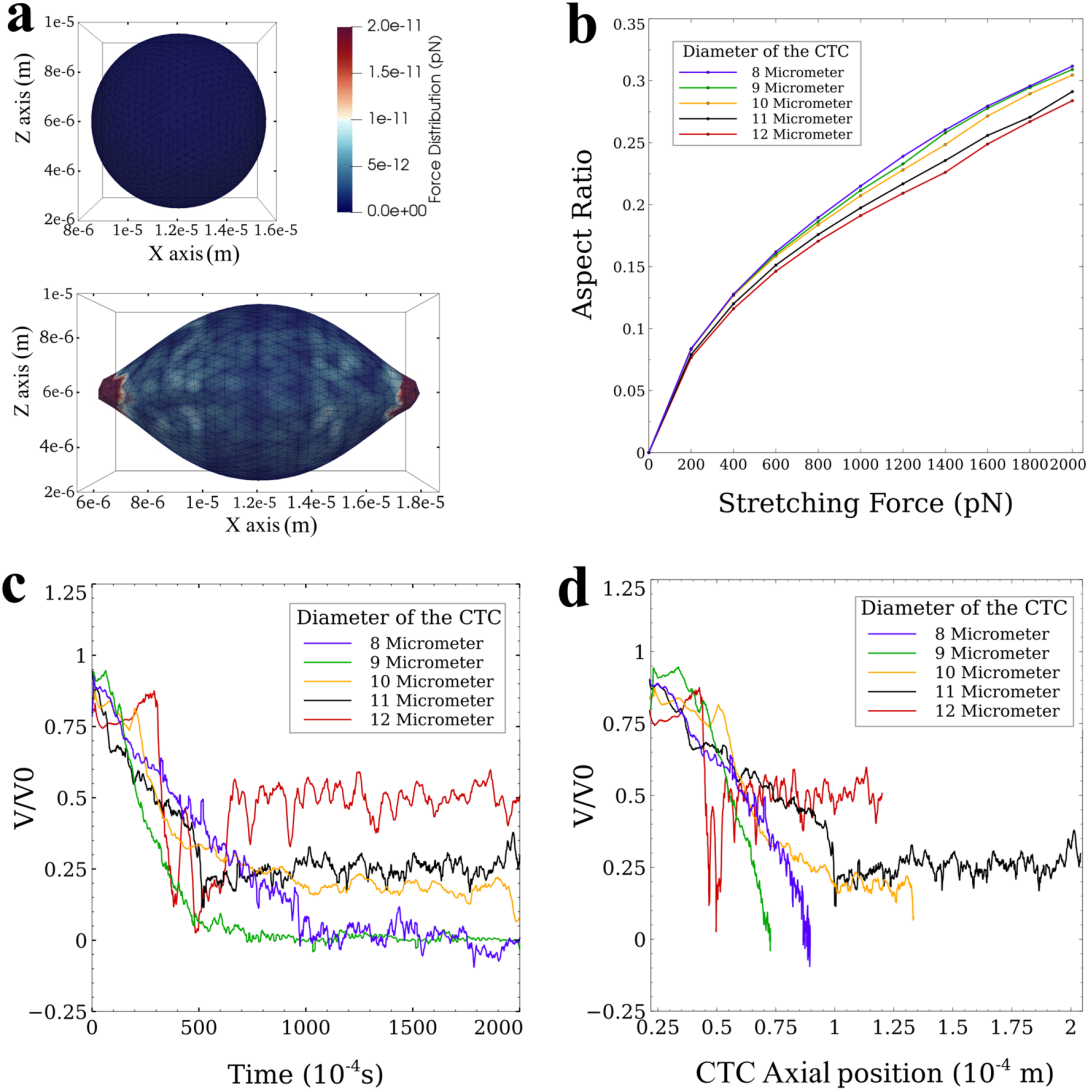
Analysis of the effect of CTC diameter on its deformation and adhesion to the endothelial cells. (**a**) Stretch test simulation on CTCs with different diameters (Supplementary Table S1 of Supporting Information). Top: undeformed CTC; Bottom: CTC under tension force; (**b**) *Aspect Ratio-Tension Force* obtained from stretch test simulation. The variation in the mechanical deformation of the CTC with diameters ranging from 8 to 12 µm is within 10 percent; (**c**) *Velocity–Time* graph of the effect of size of the CTC with 5 attached platelets on its adhesion dynamics. CTCs with diameters equal or smaller than 9 µm form a firm adhesion with the vessel wall, but larger CTCs continue their rolling motion in the microvessel. The velocity of the rolling CTC also increases for larger CTCs; (**d**) *Velocity–Axial Position* graph of the effect of the CTC size on the rolling distance. The 9-µm CTC rolls a shorter distance than the 8-µm CTC does to form firm adhesion bonds. This is due to the ratio of the CTC diameter to the diameter of the vessel (124). CTCs with diameters larger than 9 µm continue their rolling motion (no firm adhesion observed for the length of simulation).

Furthermore, we studied the effect of the diameter of CTCs on their adhesion to the vessel wall in the presence of platelets. We considered CTCs with diameters ranging from 8 to 12 μm. To investigate the effect of size of the cell on its deformation, we carried out a stretch test on CTCs of varying sizes similar to the tests performed both experimentally^45^ and computationally^46^ for RBCs. Because of the higher stiffness of CTC, the applied maximum tension force reaches 2000 pN which is tenfold of its value in testing a RBC. The results of the stretch test for different diameters are listed in Supplementary Table S1 in the Supporting Information. Figure 7a shows the force distribution on the 8-μm diameter CTC at the start (no applied force) and the end of the stretch test (with 2000 pN tension force). At the end of the tension test, the maximum force that the nodes of the cell locally bore was 20 pN and the major and minor axes lengths were 13.45 and 7.06 μm, respectively (aspect ratio of 0.31). Supplementary Video S5 displays the deformation of the 8-μm diameter CTC under 2000 pN tension force. Figure 7b shows the evolution of the aspect ratio of the cells during the tension test for all cell sizes. As this figure shows, the maximum difference between the deformations of cells in terms of aspect ratio between the largest and smallest CTC is less than 10%.

The *Velocity–Time* graph (Fig. 7c) shows the firm adhesion state (zero velocity) happened for CTCs with 5 platelets attached with diameters of 8 μm (baseline) and 9 μm but for larger CTCs, i.e. diameters ranging from 10 to 12 μm, the CTC continues its rolling motion up to 0.20 s that was simulated. Since the number of platelets is the same (5 platelets) for all the simulations, we can conclude that as the diameter of CTC increases, the CTCs start rolling faster which is due to excessive forces applied by the flow of plasma and RBCs.

One of the interesting findings of the size analysis is that the adhesion of CTC with 9 μm diameter took place more effectively, i.e. faster and in the smaller rolling distance, than CTC with a diameter of 8 μm (Fig. 7d). This can be partially due to a specific ratio of vessel diameter to CTC diameter as investigated thoroughly by Takeishi et al.^36^. The arrest of smaller cell clusters becomes more important when we consider their ability to extravasate easier through endothelial cells.

## Discussion

We showed that upon rolling of the CTC near the microvessel wall, a localized vortex is formed near the vessel wall. The velocity of the plasma flow near the vessel wall should be around 0 but because of this localized vortex, the velocity of the plasma flow in vicinity of the CTC reaches to twice of the maximum flow velocity before vortex formation. As a result, the localized vortex leads to higher shear forces around the CTC that can activate the adhesion molecules on the surrounding platelets^30^ and trigger the interactions between the CTC and the attached platelets. Activation of one platelet may lead to activation of more platelets similar to the blood clot formation process. Attachment of the activated platelets to the CTC can enhance the CTC arrest and extravasation out of the vessel wall and aggravate the metastatic outcome.

Our result quantitatively showed that the adhesion of platelets to the CTC encourages the firm adhesion of the CTC to the vessel wall and increasing the number of platelets accelerates the adhesion to the vessel wall. In other words, the adhesion of the platelets to the CTC reduces the time of the exposure of the CTC to the shear stress induced by the plasma flow which is a critical factor for the viability of CTC as demonstrated in vitro^47^ and it reduces the distance of the CTC rolling movement on endothelial cells which can harm the membrane of the CTC.

Another aspect of the interactions between platelets and CTCs that we observed in the results of the simulation is the ability of the attached platelets to reduce large deformations resulting from collisions with RBCs and the hemodynamic shear forces applied by the plasma flow. The mechanical stresses induce apoptotic cell death in CTCs^48^. Takamatsu et al. demonstrated that 55% increase of the strain on the cell surface that is induced by compressing the cell with two flat plates, decreases the viability of the cancer cells by 50%^49^. Our results quantitatively showed the remarkable role of platelets in reducing the forces applied on the CTC, reducing its deformations and preserving the integrity of the CTC thus substantially increasing the durability of the CTC^8^. As Fig. 3b illustrates, the total force exerted on the CTC membrane decreases when there is a layer of platelets around the CTC. Thus, platelets covering a CTC can significantly increase the survival rate of CTCs.

High shear stress has a critical role in the activation and adhesion of platelets to the endothelial cells. Savage et al. revealed that the elevated shear stress initiates and sustains platelet adhesion regulated by endothelial-derived von Willebrand factor expression^50^. The results of our simulation show that the area of elevated shear stress expands proportionally to the number of platelets attached to the CTC (Fig. 4a,b). The expanded area of shear stress and the higher total shear force applied on the endothelial cells indicate higher chance of CTC arrest and extravasation. The endothelial-cell shear-stress response requires platelet endothelial cell adhesion molecule-1 (PECAM-1, CD31) which can sense exerted forces by blood flow and can lead to transactivation of endothelial VEGFR2. VEGFR2 triggers conformational activation of integrins leading to the firm platelet-endothelial adhesion^51^. PECAM-1 expression is largely concentrated at junctions between adjacent cells and can regulate transendothelial migration of cells^52,53^.

Furthermore, the integrity of the vascular endothelium within the microenvironment of the attached CTC may be directly, or indirectly, affected by growth factors released from platelet α-granules, including platelet-derived growth factor (PDGF), TGFβ, EGF, and VEGFA^5,54^. Therefore, the platelets can modulate the permeability of the vessel wall and facilitate CTC extravasation. This exemplifies another means by which interference with platelet activation can worsen metastasis outcome. Since the immunosurveillance is primarily based on direct interaction of immune cells and CTCs, the platelets attached to the CTC may serve as a shield against immune assault^55^. Our results also show that attachment of platelets to softer and smaller CTCs are more dangerous and provides even easier adhesion and arrest in comparison with attachment to other CTCs (Figs. 6a,b, 7c,d). In addition, the ratio of the diameter of CTC to the diameter of the microvessel seems to have a considerable effect on the CTC adhesion (Fig. 7c,d).

There exist several experimental studies that highlight the role of platelets in the metastasis process. Gastpar et al.^22^ performed several experiments to observe the interaction between platelets and CTCs and also to test the effect of different platelet-aggregation inhibitors on the metastasis process. During the experiments on the mesentery of rats, the number of platelets was counted five minutes before and thirty minutes after CTC transplantation. They observed that the number of circulating platelets decreased drastically after CTC transplantation which is because of platelet adhesion to CTCs and attachment to the vessel wall^22,56^. Furthermore, it was reported that with the injection of platelets, the number of CTCs that adhere to the vessel wall which are observed with a fluorescent microscope^57^ increased confirming the platelet–CTC interactions. Gastpar reported that with the use of platelet-aggregation inhibitor drugs (such as aspirin, heparin, and mopidamole RA 233), the probabilities of CTC arrest, CTC extravasation, and metastatic tumor formation were decreased because these drugs inhibit platelets from adhering to CTCs^22^. Borsig et al.^21^ undertook a detailed study focusing on heparin treatment. They showed that injecting heparin can inhibit interactions of platelets with carcinoma cell-surface ligands. Therefore, CTC–platelet interaction was impaired leading to a reduction of the metastatic spreading^21^. Papa et al. suggested that using platelet decoys, modified platelets that lack aggregation and activation capacity, would inhibit the platelet-mediated pathogenic processes associated with cancer metastasis^58^.

Since the number of CTCs is substantially low in comparison to red blood cells and platelets^59^, we considered one individual cancer cell in all simulations. Future studies should evaluate the adhesion effects of platelets on CTCs when more CTCs are present in close neighborhood of one another. Moreover, since this study was performed in a straight microvessel, effects of curvature and sophisticated vasculature networks^60^ should be studied in future. The present computational study provides important evidence about the mechanisms by which platelets contribute to the metastasis process. Further investigations should be conducted to explore these mechanisms experimentally.

## Materials and methods

In microcirculation, the rheology of blood is highly dependent on the behaviour of individual cells and their interactions in the flow. We used actual physiological parameters are used to emulate the circulation of cancer cell as close as possible to the same process in the human body. Because the aim of this study was to investigate the behaviour of individual cancer cells in microcirculation, we modelled the blood as a suspension of deformable cells (RBCs, platelets, CTC) in a viscous plasma flow. We modelled the plasma as an incompressible Newtonian fluid using the lattice Boltzmann method implemented in Palabos open-source code (Ver 2.0r0)^61^. We modelled deformable cells (RBCs, platelets and CTCs) using solid Discrete Element Method (DEM)^61^ in interaction with the plasma flow via IBM^62^ using the HemoCell open-source code (version 2.0)^2,63–65^ augmented in house to allow modelling of adhesion. Please refer to the Supporting Information for details.

### Plasma flow model using Lattice Boltzmann Method

The basis of the LBM formulation, we used in this study, was proposed by Bhatnagar, Gross, and Krook (BGK)^66^ where

1 $${f}_{i}\left(\overrightarrow{x}+{\overrightarrow{e}}_{i} , t+1\right)={f}_{i}\left(\overrightarrow{x},t\right)+\frac{1}{\tau }\left({f}_{i}^{eq}\left(\overrightarrow{x},t\right)-{f}_{i}\left(\overrightarrow{x},t\right)\right).$$

The *i* index indicates the discrete velocity direction, $\overrightarrow{x}$ is the position of the particle, $t$ denotes the time, ${f}_{i}^{eq}$ defines the equilibrium distribution function, ${\overrightarrow{e}}_{i}$ is the direction of the selected velocity, and $\tau$ is the relaxation time. Using the first two moments of the distribution function, the fluid density $\rho$ and the macroscopic velocity $\overrightarrow{u}$ can be calculated at any site with the following equations:

2 $$\rho =\underset{i}{\sum }{f}_{i},$$

3 $$\overrightarrow{u}=\frac{1}{\rho }\sum_{i}{f}_{i}{\overrightarrow{e}}_{i}.$$

The equilibrium distribution function was obtained from the Boltzmann distribution:

4 $${f}_{i}^{eq}\left(\overrightarrow{x},t\right)={w}_{i}\rho \left[1+3\left({\overrightarrow{e}}_{i}.\overrightarrow{u}\right)+\frac{9}{2}{\left({\overrightarrow{e}}_{i}.\overrightarrow{u}\right)}^{2}-\frac{3}{2}{\overrightarrow{u}}^{2}\right],$$

where the ${w}_{i}$ indicates the grid dependent weight values. Based on Enskog–Chapman analysis^67^ of the limit of long wavelengths, the above-defined system can be related to Navier–Stokes equation for incompressible flows with kinematic viscosity of $\nu ={c}_{s}^{2}\left(\tau - \frac{1}{2}\right)$ and an ideal equation of state.

$p\left(\rho \right)=\rho {c}_{s}^{2}$ in which $p\left(\rho \right)$ stands for the pressure and ${c}_{s}$ is the grid-dependent speed of sound with the assumed value of $\frac{1}{\sqrt{3}}$.

### Cell deformation model using Discrete Element Method

The solid phase was modelled using a coarse-grained spectrin-link membrane model^65^ modified from a model proposed by Fedosov et al.^46^. In this approach the cell is modelled as a solid membrane discretized with triangular elements and its deformation is governed by reaction forces. Since the reaction forces originate from different features of the cells, the model assumes that for small deformations, the forces present a linear regime with different slopes. However, for large enough deformations, a quickly-diverging nonlinear term will come into effect. In the coarse-grained spectrin-link membrane model used in this work there are four types of forces each representing a specific mechanical behaviour. For more information about the Discrete Element Method used in this study, please refer to Supporting Information.

### Plasma-cell interaction model using Immersed Boundary Method

Because the fluid nodes in the LBM are on a structured Eulerian grid, whereas solid cells use Lagrangian grids, the grid of a cell may not coincide with the Eulerian fluid grid. We coupled the fluid and solid domains and modelled their interactions using the immersed boundary method (IBM)^62^ implemented and validated with an efficient parallel design in HemoCell open source code^68,69^. Different modes of cell motions (such as parachute motion, aggregation of RBCs, and margination of platelets) in HemoCell were already validated against experimental data. The fundamental assumption in IBM is the no-slip condition at the solid–fluid interface. The Lagrangian node of the cell surface ${x}_{i}\left(t\right)$ exerts the force ${F}_{i}\left(t\right)$ to the adjacent Eulerian node $X$ of the fluid based on the following equation^68,69^:

5 $$f\left(X, t\right) = \sum_{i}{F}_{i}\left(t\right) \delta \left(X-{x}_{i}\left(t\right)\right),$$

where $\delta \left(X-{x}_{i}\left(t\right)\right)$ is the discrete Dirac delta function. Then, the position of the particle is updated according to the Eulerian scheme with the following formulation:

6 $${x}_{i}\left(t+\Delta t\right)={x}_{i}\left(t\right)+{u}_{i}\left(t+\Delta t\right)\Delta t,$$

where

7 $${u}_{i}\left(t+\Delta t\right)=\sum_{i}u\left(X, t+\Delta t\right) \delta \left(X-{x}_{i}\left(t\right)\right).$$

The 1-D interpolation of the kernel functions $\phi$, provides $\delta \left(r\right)$ as: $\delta \left(r\right)=\phi \left(x\right)\phi \left(y\right)\phi \left(z\right)$. For the simplicity and compact support, the formulation of the kernel function considered in this work is:

8 $$\phi \left(r\right)=\left\{\begin{array}{c}1-\left|r\right| \left|r\right|\le 1\\ 0 \left|r\right|>1.\end{array}\right.$$

More information about the IBM method used in this work is available in Refs.^68,69^.

### Description of adhesive-dynamics model

For simulating the adhesion forces between platelets, CTC, and endothelial cells, we implemented an adhesive-dynamics model proposed by Hammer and Apte^27^. This model was originated from Bell’s model of adhesion^70^ and simulates all of the phases of the adhesion process from the unencumbered motion of the cells to firm adhesion and considers the effects of different parameters like the number of receptors, density of ligands, and fluid-flow variables such as shear rate^27^. This model has been commonly used (with some small modifications) for simulating receptor-ligand adhesion in different studies. It employs a stochastic Monte Carlo method integrated with kinetics models to simulate the formation and rupture of receptor-ligand bonds. The kinetics of the models is based on the Dembo model^71^ which formulates the rate constants as a function of distance. The forward and reverse rates for the receptor-ligand bond are calculated using the following equations:

9 $${k}_{f}={k}_{f}^{0}exp\left[-\frac{{\sigma }_{ts}{\left(l-{l}_{0}\right)}^{2}}{2{K}_{B}T}\right],$$

10 $${k}_{r}={k}_{r}^{0}exp\left[\frac{\left({\sigma }_{b}-{\sigma }_{ts}\right){\left(l-{l}_{0}\right)}^{2}}{2{K}_{B}T}\right],$$

where ${k}_{f}^{0} and {k}_{r}^{0}$ are the unstressed forward and reverse reaction rates,$l and {l}_{0}$ are the stretched and equilibrium bond lengths, ${\sigma }_{ts}$ is the spring constant in the transition state, ${\sigma }_{b}$ is the spring constant in the bonded state, ${K}_{B}$ is the Boltzmann constant, and $T$ is the absolute temperature. Because of the complexity and huge calculation cost, we skipped the tethering phase of adhesion and we focused on the adhesion process led by integrin molecules and we chose the values of the adhesive dynamics model based on that. We assumed a small value for ${\sigma }_{ts}$ in this work indicating that forward reaction is possible with ${k}_{f}^{0}=1000 {\mathrm{s}}^{-1}$ for adhesive molecules with considerable distance^27^. This assumption is valid because we ignored the initial phase of adhesion starting with the tethering process that is led by selectin molecules.

The force applied by the receptor-ligand bond is assumed to follow the Hookean spring model,

11 $${F}_{bond}={\sigma }_{b}\left(l-{l}_{0}\right)\hat{e},$$

where ${\sigma }_{b}$ is the spring constant and $\hat{e}$ is the direction of exerted adhesive force of each bond that depends on the position of the adhesive molecules (defined by nodes) on the surface of CTC, platelets, or the endothelial cells. For example the time-varying vector of ${\overrightarrow{e}}_{{f}_{PLT-CTC}}= {\overrightarrow{O}}_{CTC}- {\overrightarrow{O}}_{PLT}$ determines the direction of the adhesive force between platelet and a CTC where ${\overrightarrow{e}}_{{f}_{PLT-CTC}}$ is the direction of the force exerted on a platelet by an adhesive bond between platelet and a CTC, ${\overrightarrow{O}}_{CTC}$ is the position of the adhesive molecule on the CTC, and ${\overrightarrow{O}}_{PLT}$ is the position of the adhesive molecule on the platelet.

The probability of the formation of a new bond (${P}_{f}$) and rupture of an existing bond (${P}_{r}$) during the time interval $\Delta t$ is calculated using the following equations:

12 $${P}_{f}=1-exp\left(-{k}_{f}\Delta t\right),$$

13 $${P}_{r}=1-exp\left(-{k}_{r}\Delta t\right).$$

For reducing the computational cost, we used a new method in this study for applying the results of the probability function on the model. Since the time interval chosen for probability calculation is very small (0.1 $\mathrm{\mu s}$) relative to the simulation time, we defined a new parameter, the “effective force”, to integrate the force of each adhesion bond and the probability of formation and rupture of the bond. The effective force (${F}_{eff}$) of each bond equals the product of the calculated force of the bond (${F}_{bond}$) multiplied by the effective probability,

14 $${F}_{eff}= {P}_{eff}\times {F}_{bond}.$$

Given the small time step for calculating the probability and force of each receptor-ligand bond, the variables of the probability function do not significantly change and we can assume that ${P}_{{f}_{t}}\cong {P}_{{f}_{t-1}}$ and ${P}_{{r}_{t}}\cong {P}_{{r}_{t-1}}$ for formation and rupture of the bonds respectively. Therefore, for each bond we have:

15 $${P}_{eff}={P}_{f}\left(1-{P}_{r}\right).$$

After calculating the effective force using the effective probability function on each bond, probabilities ${P}_{f}$ and ${P}_{r}$ are updated at each time step. Compared to the original algorithm^27^, in addition to reducing the computational cost, this approach also increases the stability of the simulation without changing the outcome by preventing abrupt appearance and disappearance of forces at each time step. In this work, we only focused on the receptor-ligand type of adhesion between CTC, platelets, and endothelial cells, therefore we considered the same adhesion properties for all of them. The information about the constants used in this model can be found in the Supplementary Table S2 of Supporting Information.

### Geometry and boundary conditions

The computational domain was considered to be a cylindrical microvessel with a diameter of 20 μm and a length of 40 μm and periodic boundary conditions in the axial direction at the base faces of the cylinder. The microvessel wall was considered to be smooth and rigid, which are acceptable assumptions for small blood vessels, with no-slip boundary conditions imposed with the bounce-back algorithm^44^. Poiseuille flow with Reynolds number of 0.025 similar to^44^, was considered for initializing the simulation and the physical volume of a lattice unit was equal to 0.5 × 0.5 × 0.5 μm^3^.

We considered the CTC to be a sphere with a diameter of 8 μm^42,43^ and generated the mesh for it with 1382 vertices using the Mefisto algorithm in SALOME 9.3.0.. We employed the same software and the same algorithm for refining the mesh of an existing platelet geometry to have 1148 vertices. For more details about the geometry and the boundary conditions, please refer to Supporting Information.

### Sensitivity to simulation time span

To study the effect of the simulation time on our results and conclusions, we continued the simulations of two cases (0-platelets and 15-platelets attached to the CTC) for additional 2 million time steps to make sure our final results do not change after the simulation time. We observed that for 0-platelets attached to the CTC, the CTC continues its rolling motion without any significant changes in its velocity (stayed similar to the Velocity–Time graph of 0-platelets in Fig. 2a). For the case with 15-platelets attached to the CTC, the firm adhesion between the CTC and the vessel wall and the location of the CTC stayed unchanged. Additionally, the aspect ratio of the CTC in both cases increased less than 10%. Based on these results we can conclude that the final results of our model are stable and not sensitive to the simulation time span.

## Supplementary Information

Supplementary Information.

Supplementary Video 1.

Supplementary Video 2.

Supplementary Video 3.

Supplementary Video 4.

Supplementary Video 5.
