# Supplementary material for: Interactions of platelets with circulating tumor cells contribute to cancer metastasis
Source: Sci Rep. 2021 Jul 29;11:15477. doi: 10.1038/s41598-021-94735-y (PMC8322323; doi:10.1038/s41598-021-94735-y)
Supplement: Supplementary file 1 — Supplementary Information. [file 41598_2021_94735_MOESM1_ESM.docx]

# Supporting Information

## Cell deformation model using Discrete Element Method

In the coarse-grained spectrin-link membrane model used in this work there are four types of forces each representing a specific mechanical behaviour.

1. The link force acts along the links and represents the reaction to stretching and compression of the underlying spectrin-network beneath the links. The formulation of the force presents a linear part corresponding to smaller deformations and a fast-diverging nonlinear part which represents the limits of the material as the stretch approaches the persistence-length.
   $F_{link}=-\frac{k_{l}dL}{\varpi}\left[ 1+\frac{1}{\tau_{l}^{2}-dL^{2}} \right]$ (1)
   where $dL=\frac{L_{i}-L_{0}}{L_{0}}$ is the normal strain (relative deviation from the equilibrium length of the surface element ($L_{0}$)), $\varpi$ is the persistence-length of a spectrin filament that equals to 7.5 nm ^1^, and $\tau_{l}=3.0$0 is the relative expansion ratio at which the spectrin-network reaches its persistence length ^2^.
2. The bending force acts between two adjacent cell surface elements and represents the membrane reaction force due to the underlying cytoskeleton and the non-zero thickness of the spectrin-network. On each surface, the bending force is in the direction normal to the surface.
   $F_{bend}=-\frac{k_{b}d\theta}{L_{0}}\left[ 1+\frac{1}{\tau_{b}^{2}-d\theta^{2}} \right]$ (2)
   where $d\theta=\theta_{i}-\theta_{0}$, and $\tau_{b}=\frac{\pi}{6}$ is the limiting angle. Using the micropipette aspiration images ^3^, $\tau_{b}$ was calibrated for this problem and it was chosen to prevents unrealistic sharp surface edges.
3. The local surface conservation force represents the reaction of the membrane and the spectrin-network to stretching and compression. The local surface conservation force acts locally on each surface element and is applied equally to each of the three vertices of cell surface triangles and points toward the centroid of the triangles ^2^.

$F_{area}=-\frac{k_{a}dA}{L_{0}}\left[ 1+\frac{1}{\tau_{a}^{2}-dA^{2}} \right]$ (3)
where $dA=\frac{A_{i}-A_{0}}{A_{0}}$ is the relative deviation from the initial area and $\tau_{a}=0.30$ is the limiting factor to prohibit surface area changes more than 30% ^2^. For $dA$ near 30%, the area force gets large enough to stop further expansion of the surface area. Large changes in the surface area can lead to permanent damages to the cell membrane ^4^.

1. The volume conservation force acts globally on all nodes of the cell and is responsible to maintain the quasi-incompressibility of the cell. The volume conservation force is toward the normal of the surface of each surface triangle.

$F_{volume}=-\frac{k_{v}dV}{L_{0}}\left[ \frac{1}{\tau_{v}^{2}-dV^{2}} \right]$ (4)
where $dV=\frac{V_{i}-V_{0}}{V_{0}}$ is the relative deviation from initial volume and $\tau_{v}=0.01$ is the limiting factor to resist changes in the cell volume. The volume of the cell should be preserved because of the law of conservation of mass ^2^.

$k_{l}, k_{b}, k_{a}, k_{v}$ in the above equations are the parameters chosen to satisfy the mechanical single-cell experimental results. The above constitutive model described by Equations (1) to (4) was validated for the RBC and the values for parameters were reported using optical tweezers experiment ^5^. This model was also used for platelets with rough estimates of the mechanical properties ^2^. In the present work, we refined the mechanical properties of the platelets based on the results of the work of Haga et al. ^6^.

We also modelled CTCs using the above constitutive equations with the parameter values provided in Table S2. The mechanical properties of cancer cells differ from one type of cancer to another and even for a specific type of cancer, properties of cancer cells are different in an individual patient depending on the malignancy of the cancerous cells ^7,8^. Generally, metastatic tumor cells are softer than normal tumor cells enabling them to deform more efficiently ^8^. Softer CTCs can deform and as a result, the contact area between them and the vessel wall expands which favours firm adhesion. Fedosov and Gompper^9^ studied the movement of white blood cells in microvessels using a coarse-grained spectrin-link membrane model, similar to one used in the present work, and considered white blood cells to be one order of magnitude stiffer than RBCs ^9^. Based on the similarities between the mechanical properties of white blood cells and CTCs ^10,11^, and the work of Fedosov and Gompper^9^ on white blood cells, we considered the stiffness of the CTCs in our model to be about 10 times of the stiffness of the RBCs. This is consistent with the stiffness that Lenarda et al. ^12^, used for CTCs. Additionally, we studied adhesion of the softer and stiffer CTCs and discussed the stiffness effects in the Results section of the main manuscript.

## Time step

There are three types of time steps in our model: 1. fluid field (LBM) 2. fluid-solid coupling (IBM) 3. cell model (DEM) which includes about 4000 equations per cell ^13^. The DEM is the most computationally expensive one among the three. When the maximum force at each time step in the domain is below a lower limit set by the simulation, the time step for updating DEM can be increased ^13^. When the maximum force is between the lower limit and upper limit, the time step should not be changed and finally, when the maximum force is above the upper limit of the maximum force generated within the system, the time step for updating the material model should be decreased for providing an accurate and stable outcome. These limits are fine-tuned based on the geometry of the problem to guarantee a balance between performance and numerical stability ^13^. With this approach, the performance of the computation can be optimized because the computation time is strategically spent on the specific time intervals of the simulation that need finer time steps to reach an accurate and stable solution ^14^. In general $dt_{LBM}<dt_{IBM}<dt_{DEM}$ but in this work, because of the relatively large deformations and strong adhesion forces, we set both time steps of updating the DEM model of cells and calculation of IBM equal to the time step of the LBM. The time step for the LBM was set to 0.1 μs which is a common value in similar works ^15,16^. Each simulation ran for 2,000,000 time steps which is equal to 0.2 seconds. The length of the simulation time is in order of similar works on cell adhesion ^16,17^ and was calibrated to provide enough time window for the CTC to adhere to the vessel wall and to reach a stable condition.

## Geometry and Boundary Conditions

HemoCell (version 2.0) does not have any collision detector function for solids and all the solid-solid interactions (cell-cell or cell-wall) are handled with the IBM solver and by the means of the lubrication effect of the fluid flow and therefore there was no repulsive force in the peripheral boundaries and the cells went out of the computational domain. We added a collision detector function that works in the peripheral boundaries and applies a repulsive force proportional to the momentum of the cell impacting the endothelium. In our simulations, we assumed the vessel wall to be rigid and did not model its deformation, however, and owing to the fact that the impact always involves energy loss ^18^ and little rebounding of particles happen in in plasma flow ^19^, we chose the value of 0 for the cell-wall coefficient of restitution.

The initial position of the cells in the simulation was generated randomly using the PackCells toolbox of HemoCell. We generated the initial positions for 1 CTC and 17 RBCs equivalent to a hematocrit of 12% which is a physiological value in microcirculation. Since the margination movement of the CTC is quite similar to the white blood cell and it is widely studied in the literature ^9–11,20^, at the beginning of the simulation, we intentionally put platelets near the CTC and put the CTC-platelet complex near the vessel wall to decrease the simulation time and to focus on the effect of the platelets on the CTC adhesion to the vessel wall. Figure S1-a of the Supporting Information shows a snapshot of the computational domain at t=0 including the streamlines of the plasma flow, RBCs, and a CTC. Additionally, Figure S1-b shows the velocity profile obtained at t=0. The seeds of the streamlines were uniformly distributed on the line source located at (x, y, z) = (10, 0:20, 10). The velocity profiles in the Figure S1-c shows consistency between the Poiseuille flow generated with LBM method in this study and the classical Poiseuille parabolic profile which can be found in ^21^.

In the present work we implemented the *tube hematocrit* which is defined as the instantaneous volume fraction of red blood cells in the microvessel segment with known diameter and length^22^. Based on the experimental data, the tube hematocrit in the microvessels can vary depending on the curvature and geometry of the microvessel and the instantaneous positions of red blood cells^23^. We considered the hematocrit of 12% for all the simulations according to the value of 12.10 ± 5.32% reported experimentally in microvessels^22^. Because the effects of different ranges of hematocrit on the flow of CTCs in the microvessel were studied in the past^11^, we did not exhaustively studied them here. In the current study, we focused on the number of platelets and its effect on the adhesion of CTCs and investigated the effects of varying hematocrit by ±2% about the average 12% on adhesion characteristics of a CTC surrounded by 5 platelets. As Figure S2 shows, 2% variation in the hematocrit level did not lead to remarkable difference in the adhesion behaviour of the CTC.

The simulations were done on the Niagara supercomputer at the SciNet HPC Consortium with Intel Skylake cores (2.4GHz, AVX512). We employed a total of 40 cores (that is the optimum number of cores considering the size of the domain) for each simulation parallelized using MPI. Each simulation took between 24 to 36 hours to get completed depending on the number of platelets in the simulation.

^
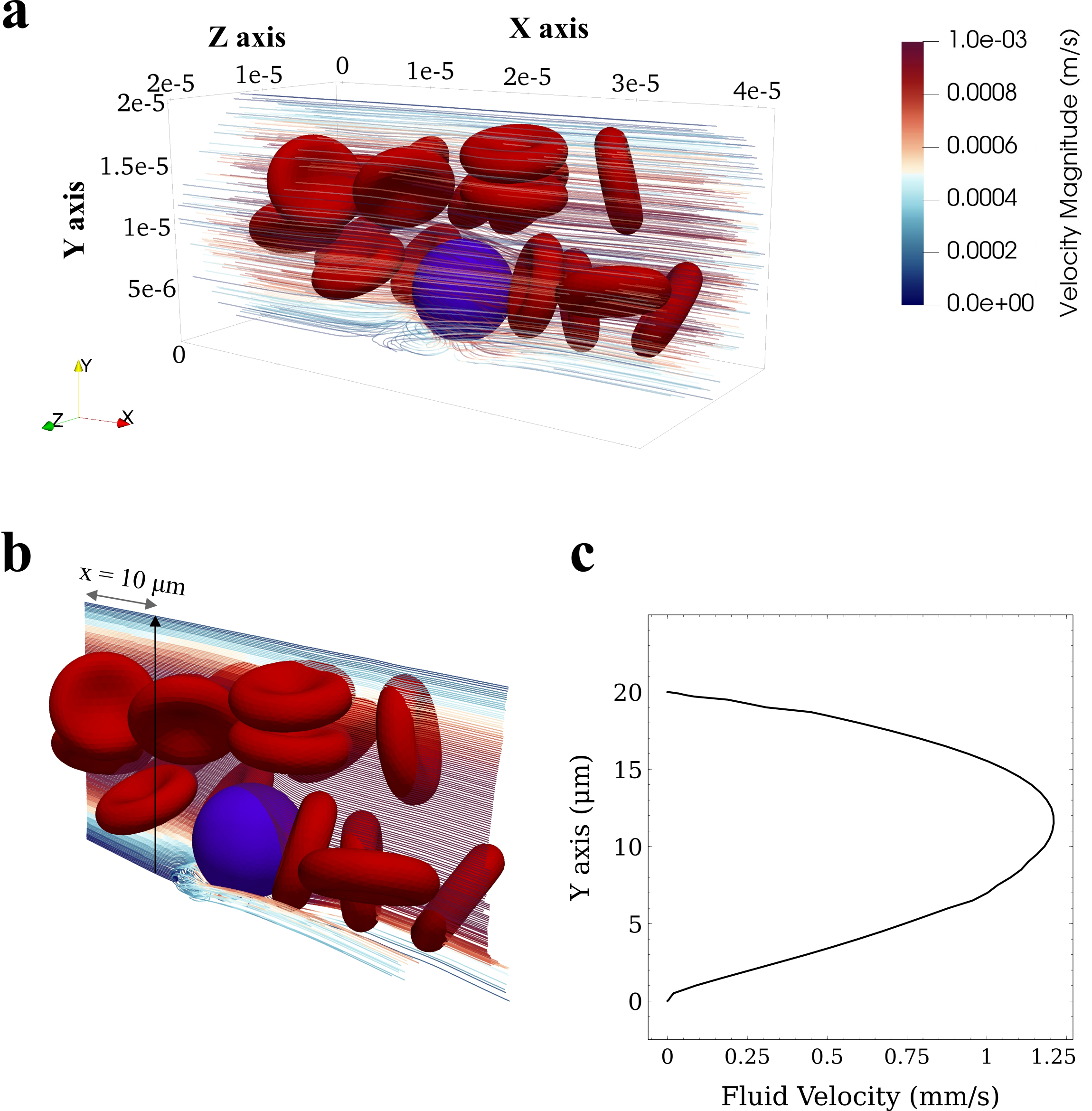
^

**Figure S1-** a) Snapshot of the computational domain including the red blood cells (red), one CTC (blue), and the plasma flow (visualized by streamlines coloured with the velocity magnitude); b) Streamlines that pass a diameter of the microvessel (black vertical line) ; c) The velocity profile of the plasma at x = 10 μm consistent with Poiseuille flow. (Prepared in Paraview v5.7, [www.paraview.org](http://www.paraview.org)).


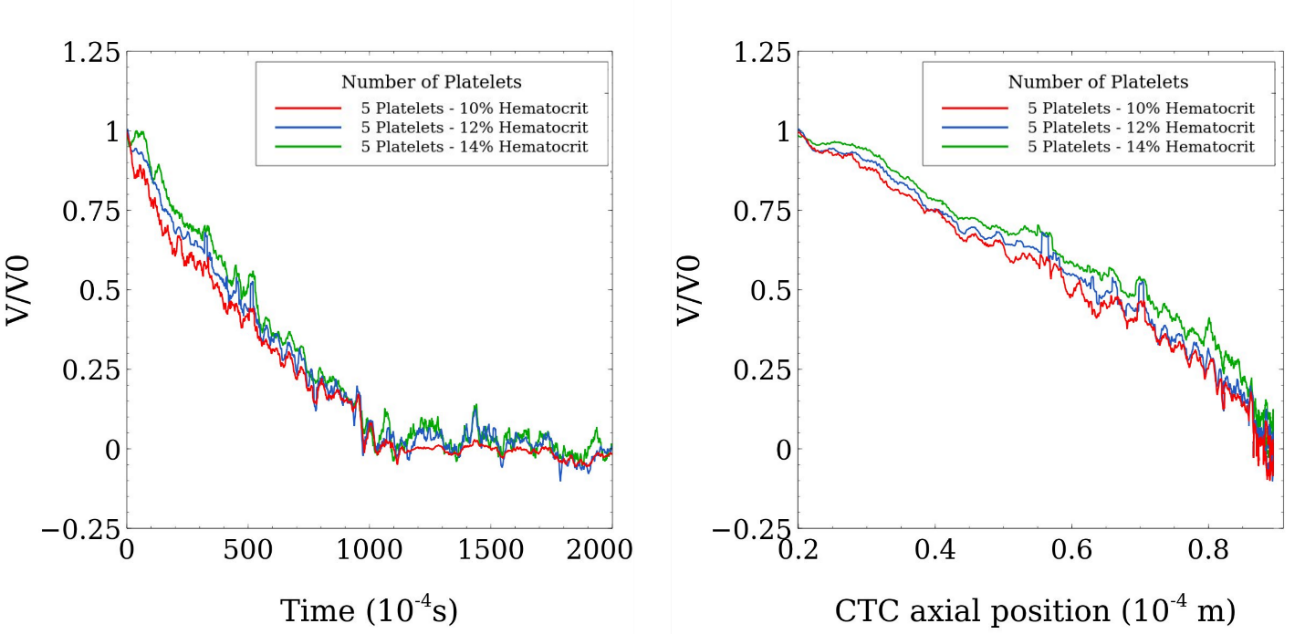


**Figure S2**- The effect of 2% variation in the hematocrit on the Velocity-Time and Velocity-Axial Position graphs of a CTC surrounded by 5 platelets. Adhesion behaviour of the CTC in terms of time and distance to form firm adhesion did not remarkably altered by changing the hematocrit level.

**Table S1- Results of the stretch test of CTCs with different diameters**


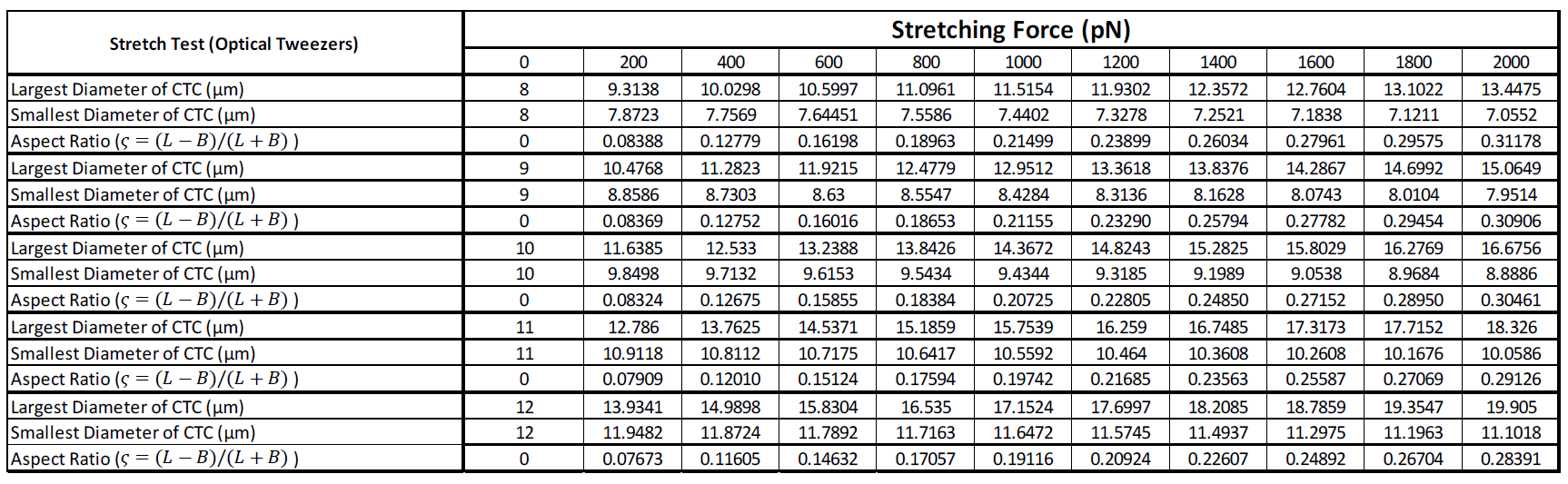


**Table S2- Parameters used in the model**

| Parameter | Definition | Physical value | Reference |
| --- | --- | --- | --- |
| $T$ | Absolute temperature | $310 K$ | ^24^ |
| $k_{f}^{0}$ | Unstressed forward reaction rate | $1000 s^{-1}$ | ^25^ |
| $k_{r}^{0}$ | Unstressed reverse reaction rate | $1 s^{-1}$ | ^26^ |
| $k_{l_{\mathrm{CTC}}}$ | Link force coefficient | 400 | ^9,12^ |
| $k_{b_{\mathrm{CTC}}}$ | Bending force modulus | 800 $K_{B}T$ | ^9,12^ |
| $k_{a_{\mathrm{CTC}}}$ | Local area conservation coefficient | 50 | ^9,12^ |
| $k_{v_{\mathrm{CTC}}}$ | Volume conservation coefficient | 200 | ^9,12^ |
| $\sigma_{ts}$ | Transition state spring constant | ${10}^{-9}\frac{N}{m}$ | ^27^ |
| $\sigma_{b}$ | Bond spring constant | ${10}^{-3}\frac{N}{m}$ | ^28,29^ |
| $l_{0}$ | Equilibrium bond length in adhesive dynamics model | $20 nm$ | ^24^ |
| $H_{c}$ | Cut-off length for bond formation | $100 nm$ | ^12^ |
| $K_{B}$ | Boltzmann constant | $1.38\times{10}^{-23} J/K$ | ^30^ |
| $\Delta t$ | Time interval | ${10}^{-7} s$ | ^15,16^ |
| $Re$ | Reynolds number | 0.025 | ^12^ |
| $\Delta x$ | Fluid lattice resolution | $0.5 \mu m$ | ^12^ |
| $D_{cp}$ | Microvessel diameter | 20 $\mu m$ | ^12^ |
| $L_{cp}$ | Length of the periodic microvessel domain | 40 $\mu m$ | ^12^ |
| $H_{t}$ | Hematocrit | 12% | ^23^ |
| $\nu$ | Kinematic viscosity of plasma | $1.2 \times{10}^{-6}\frac{m^{2}}{s}$ | ^12^ |
| $\mu$ | Dynamic viscosity of plasma | $1.2 mPa.s$ | ^31,32^ |
| $r_{CTC}$ | CTC baseline radius | $4 \mu m$ | ^33^ |

**Videos:**

**Video S1:** Rolling motion of the CTC with no platelets attached. (Prepared in Paraview v5.7, [www.paraview.org](http://www.paraview.org)).

**Video S2:** Vortex formation upon rolling motion of the CTC. (Prepared in Paraview v5.7, [www.paraview.org](http://www.paraview.org)).

**Video S3:** Firm adhesion of the CTC with 5 attached platelets. (Prepared in Paraview v5.7, [www.paraview.org](http://www.paraview.org)).

**Video S4:** Firm adhesion of the CTC with 10 attached platelets. (Prepared in Paraview v5.7, [www.paraview.org](http://www.paraview.org)).

**Video S5:** Stretch test on CTC with 2000 pN tension force. (Prepared in Paraview v5.7, [www.paraview.org](http://www.paraview.org)).

# References of the supporting information

1. Li, J., Dao, M., Lim, C. T. & Suresh, S. Spectrin-Level Modeling of the Cytoskeleton and Optical Tweezers Stretching of the Erythrocyte. *Biophysical Journal* **88**, 3707–3719 (2005).

2. Závodszky, G., van Rooij, B., Azizi, V. & Hoekstra, A. Cellular Level In-silico Modeling of Blood Rheology with An Improved Material Model for Red Blood Cells. *Front. Physiol.* **8**, (2017).

3. Mohandas, N. & Evans, E. Mechanical Properties of the Red Cell Membrane in Relation to Molecular Structure and Genetic Defects. *Annu. Rev. Biophys. Biomol. Struct.* **23**, 787–818 (1994).

4. Li, F., Chan, C. U. & Ohl, C. D. Yield Strength of Human Erythrocyte Membranes to Impulsive Stretching. *Biophysical Journal* **105**, 872–879 (2013).

5. Suresh, S. *et al.* Connections between single-cell biomechanics and human disease states: gastrointestinal cancer and malaria. *Acta Biomaterialia* **1**, 15–30 (2005).

6. Haga, J. H., Beaudoin, A. J., White, J. G. & Strony, J. Quantification of the Passive Mechanical Properties of the Resting Platelet. *Annals of Biomedical Engineering* **26**, 268–277 (1998).

7. Yu, H., Mouw, J. K. & Weaver, V. M. Forcing form and function: biomechanical regulation of tumor evolution. *Trends in Cell Biology* **21**, 47–56 (2011).

8. Guck, J. *et al.* Optical Deformability as an Inherent Cell Marker for Testing Malignant Transformation and Metastatic Competence. *Biophysical Journal* **88**, 3689–3698 (2005).

9. Fedosov, D. A. & Gompper, G. White blood cell margination in microcirculation. *Soft Matter* **10**, 2961–2970 (2014).

10. Mitchell, M. J. & King, M. R. Computational and Experimental Models of Cancer Cell Response to Fluid Shear Stress. *Front. Oncol.* **3**, (2013).

11. Takeishi, N., Imai, Y., Yamaguchi, T. & Ishikawa, T. Flow of a circulating tumor cell and red blood cells in microvessels. *Physical Review E* **92**, (2015).

12. Lenarda, P., Coclite, A. & Decuzzi, P. Unraveling the Vascular Fate of Deformable Circulating Tumor Cells Via a Hierarchical Computational Model. *Cel. Mol. Bioeng.* **12**, 543–558 (2019).

13. Zavodszky, G., van Rooij, B., Azizi, V., Alowayyed, S. & Hoekstra, A. Hemocell: a high-performance microscopic cellular library. *Procedia Computer Science* **108**, 159–165 (2017).

14. Závodszky, G. & Paál, G. Validation of a lattice Boltzmann method implementation for a 3D transient fluid flow in an intracranial aneurysm geometry. *International Journal of Heat and Fluid Flow* **44**, 276–283 (2013).

15. Wu, T.-H. & Qi, D. Investigation of shear rates of rolling adhesion on leukocytes with bending of microvilli. *Phys. Rev. Fluids* **4**, 063101 (2019).

16. Wang, W., Mody, N. A. & King, M. R. Multiscale model of platelet translocation and collision. *Journal of Computational Physics* **244**, 223–235 (2013).

17. Mody, N. A., Lomakin, O., Doggett, T. A., Diacovo, T. G. & King, M. R. Mechanics of Transient Platelet Adhesion to von Willebrand Factor under Flow. *Biophysical Journal* **88**, 1432–1443 (2005).

18. Meriam, J. L. & Kraige, L. G. *Engineering Mechanics: Dynamics*. (John Wiley & Sons, 2012).

19. Hyakutake, T., Tominaga, S., Matsumoto, T. & Yanase, S. Numerical Study on Flows of Red Blood Cells With Liposome-Encapsulated Hemoglobin at Microvascular Bifurcation. *Journal of Biomechanical Engineering* **130**, 011014 (2008).

20. Takeishi, N., Imai, Y., Nakaaki, K., Yamaguchi, T. & Ishikawa, T. Leukocyte margination at arteriole shear rate. *Physiological Reports* **2**, e12037 (2014).

21. Koplik, J., Banavar, J. R. & Willemsen, J. F. Molecular dynamics of Poiseuille flow and moving contact lines. *Phys. Rev. Lett.* **60**, 1282–1285 (1988).

22. Desjardins, C. & Duling, B. R. Microvessel hematocrit: measurement and implications for capillary oxygen transport. *American Journal of Physiology-Heart and Circulatory Physiology* **252**, H494–H503 (1987).

23. Pries, A. R., Neuhaus, D. & Gaehtgens, P. Blood viscosity in tube flow: dependence on diameter and hematocrit. *American Journal of Physiology-Heart and Circulatory Physiology* **263**, H1770–H1778 (1992).

24. Chang, K.-C. & Hammer, D. A. Influence of Direction and Type of Applied Force on the Detachment of Macromolecularly-Bound Particles from Surfaces. *Langmuir* **12**, 2271–2282 (1996).

25. Bhatia, S. K., King, M. R. & Hammer, D. A. The State Diagram for Cell Adhesion Mediated by Two Receptors. *Biophysical Journal* **84**, 2671–2690 (2003).

26. Chang, K.-C., Tees, D. F. J. & Hammer, D. A. The state diagram for cell adhesion under flow: Leukocyte rolling and firm adhesion. *Proceedings of the National Academy of Sciences* **97**, 11262–11267 (2000).

27. Hammer, D. A. & Apte, S. M. Simulation of cell rolling and adhesion on surfaces in shear flow: general results and analysis of selectin-mediated neutrophil adhesion. *Biophysical Journal* **63**, 35–57 (1992).

28. Dembo, M., Torney, D. C., Saxman, K., Hammer, D. & Murray, J. D. The reaction-limited kinetics of membrane-to-surface adhesion and detachment. *Proceedings of the Royal Society of London. Series B. Biological Sciences* **234**, 55–83 (1988).

29. Takeishi, N. *et al.* Cell adhesion during bullet motion in capillaries. *American Journal of Physiology-Heart and Circulatory Physiology* **311**, H395–H403 (2016).

30. Daussy, C. *et al.* Direct Determination of the Boltzmann Constant by an Optical Method. *Phys. Rev. Lett.* **98**, 250801 (2007).

31. Dabagh, M. & Randles, A. Role of deformable cancer cells on wall shear stress-associated-VEGF secretion by endothelium in microvasculature. *PLOS ONE* **14**, e0211418 (2019).

32. Késmárky, G., Kenyeres, P., Rábai, M. & Tóth, K. Plasma viscosity: A forgotten variable. *Clinical Hemorheology and Microcirculation* **39**, 243–246 (2008).

33. Suresh, S. Biomechanics and biophysics of cancer cells. *Acta Biomaterialia* **3**, 413–438 (2007).
